# Fabrication, Microstructures and Sensor Applications of Highly Ordered Electrospun Nanofibers: A Review

**DOI:** 10.3390/ma16093310

**Published:** 2023-04-23

**Authors:** Jing Chen, Fei Rong, Yibing Xie

**Affiliations:** 1 School of Chemistry and Chemical Engineering, Southeast University, Nanjing 211189, China; 2 Southeast University—Monash University Joint Graduate School (Suzhou), Suzhou 215123, China; 3 School of Biological Sciences and Medical Engineering, Southeast University, Nanjing 211189, China

**Keywords:** electrospun nanofibers, highly ordered, sensor application, fabrication, microstructure

## Abstract

The review summarizes the fabrication, microstructures, and sensor applications of highly ordered electrospun nanofibers. In the traditional electrospinning process, electrospun nanofibers usually have disordered or random microstructures due to the chaotic oscillation of the electrospinning jet. Different electrospinning methods can be formed by introducing external forces, such as magnetic, electric, or mechanical forces, and ordered nanofibers can be collected. The microstructures of highly ordered nanofibers can be divided into three categories: uniaxially ordered nanofibers, biaxially ordered nanofibers and ordered scaffolds. The three microstructures are each characterized by being ordered in different dimensions. The regulation and control of the ordered microstructures can promote electrospun nanofibers’ mechanical and dielectric strength, surface area and chemical properties. Highly ordered electrospun nanofibers have more comprehensive applications than disordered nanofibers do in effect transistors, gas sensors, reinforced composite materials and tissue engineering. This review also intensively summarizes the applications of highly ordered nanofibers in the sensor field, such as pressure sensors, humidity sensors, strain sensors, gas sensors, and biosensors.

## 1. Introduction

Nanomaterials have at least one dimension at the nanoscale. Nanomaterials consist of zero-dimensional nanoparticles and quantum dots, one-dimensional nanofibers and nanotubes, two-dimensional nanosheets and nanofilms, and a three-dimensional architecture [1,2,3]. Nanofibers have received particular attention for their many properties, including their large surface area, porosity, ease of manufacture, good chemical, physical and mechanical properties, flexibility, and possible control over morphology [4,5,6,7,8]. Up to now, there have been various nanofiber fabrication technologies, such as drawing [9,10], spinning [11], coating [12,13], printing [14,15], etc. Electrospinning is one of the most employed techniques in the current strategies for fabricating nanofibers [16,17,18,19]. On the other hand, the nanostructure has high effect on the properties of nanomaterials. Well-controlled and highly-ordered nanostructures could contribute to promoting the performance of functional nanomaterials [20,21,22]. 

**Figure 1:**
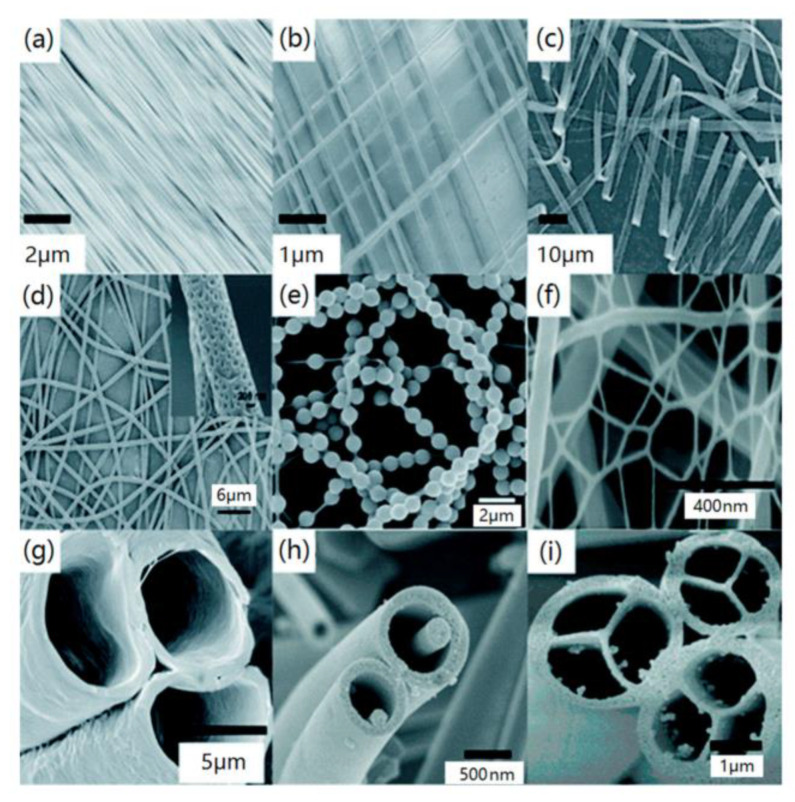
Various microstructures of electrospun nanofibers: (**a**) uniaxially ordered fiber, (**b**) biaxially ordered fiber, (**c**) ribbon-like fiber, (**d**) multi-hole fiber, (**e**) necklace-like fiber, (**f**) reticular fiber, (**g**) hollow fiber, (**h**) coaxial fiber, and (**i**) multi-channel fiber. Reproduced with permission from ref. [28].

Figure 1 shows the various microstructures of electrospun nanofibers. Nanofibers fabricated by electrospinning have small diameters, highly long lengths, large surfaces per unit mass and small pore sizes. Nanofibers can be obtained in various forms, such as uniaxially and biaxially ordered fibers [23], ribbon-like fibers [24], multi-hole fibers, necklace-like fibers [25], reticular fibers, hollow fibers, coaxial fibers [26] and multi-channel fibers [27,28]. In most cases, the microstructures of nanofibers fabricated by electrospinning are disordered because of the chaotic oscillation of the electrospinning jet. However, highly ordered nanofibers such as those in Figure 1a,b can be contained by improving the traditional electrospinning method. Depending on their differences in dimensionality, ordered nanofibers can be divided into three types. The first is uniaxially ordered, characterized by a structure in which the fibers are ordered in one dimension. The second is biaxially ordered, characterized by the ordered arrangement of fibers in two dimensions. The final one is the ordered scaffold, which is ordered and neatly aligned in all three dimensions. They also differ in their properties. Biaxially ordered fibers have the advantages of ordered fibers in more dimensions than uniaxially ordered fibers. They also differ in their properties. Uniaxially ordered fibers have the best mechanical and electrical properties in the fiber direction, while biaxially ordered fibers have more acceptable properties in both the longitudinal and transverse directions [23]. Klabukov et al. [29] prepared a triple-layer bile duct scaffold with an inner layer of polycaprolactone (PCL) and an outer layer of a copolymer of D,L-lactide and glycolide or a copolymer of L-lactide and ε-caprolactone using electrospinning. They found experimentally that the transverse and longitudinal strength of the multi-layer tubular scaffold was comparable to that measured for decellularized bile ducts. This demonstrates the unique property of electrospun scaffolds with predictable mechanical properties.

Different types of ordered fibers have their unique advantages in different fields. Uniaxially ordered nanofibers are the most common and have many applications among ordered nanofiber microstructures. Xia et al. [30] combined electrospinning and annealing to prepare uniaxially ordered Ba_0.8_SR_0.2_TiO_3_ (BST) nanofiber arrays. The humidity sensor made from this uniaxially ordered fiber array exhibited fast response recovery characteristics at room temperature, which demonstrates the potential application of the sensor in detecting environmental humidity. The biaxially ordered structure has a unique advantage in air filtration. Cheng et al. [31] prepared electrospun nanofiber membranes with excellent spinning performance, hydrophobicity and polyacrylonitrile (PAN) stability. They constructed disordered nanofiber membranes, standard-ordered nanofiber membranes and cross-ordered nanofiber membranes, verifying that the ordered structure can significantly reduce the pressure drop of fiber membranes while maintaining filtration efficiency. In the cross-ordered model, the staggered orientation of the pores not only facilitates airflow but also traps particles more effectively, resulting in a high filtration efficiency of 63% and an intermediate pressure drop of 65 Pa. In supercapacitor applications, the ordered large mesopores formed by the cross-stacking of nanofibers facilitate ion transport and electrolyte penetration and retention [32,33]. Ordered scaffolds can mimic specific morphologies of collagen scaffolds in specific tissues. Alexander et al. [34] fabricated highly ordered collagen nanofibers for scaffold production using direct-write electrospinning. Collagen single fibers with high linearity and controlled fiber placement were obtained by adjusting the acetic acid concentration, relative humidity and voltage. The results demonstrated that the constructed ordered scaffolds could be applied to prepare electrospun pure collagen scaffolds with a specific morphology. Zhang et al. [35] used melt electrospinning writing to fabricate spatially heterogeneous scaffolds with well-defined, non-uniform pore properties and proposed a comprehensive analytical model to design and analyze spatially heterogeneous scaffolds. The results demonstrate the potential applications of the novel MEW heterogeneous scaffolds serve as multiplex biological platforms to study the structure–function-biological cell performance.

The Increased orderliness of electrospun nanofibers will improve the fibers’ mechanical strength, dielectric strength, surface area and chemical properties. Isaac et al. [36] discovered improvements in mechanical and dielectric strength with nanofibers with an increased degree of order. Improvements in molecular orientation [37] and system configuration for improved performance due to fiber elongation around the rotating mandrel [38] can affect mechanical and dielectric properties. Liu et al. [39] used electrochemical methods to analyze dye adsorption on vertically ordered carbon nanofiber arrays coated with TiO_2_ nanoneedles in dye-sensitized solar cells. The experimental results show that vertically ordered nanostructures have a surface roughness of 90.6, which can provide a larger effective surface area for dye adsorption. Liu et al. [40] spun PCL nanofibers onto polyglycolic acid microfibers by the electrospinning method to obtain a core–shell fiber structure with controllable surface morphology. They characterized the hydrophilicity of the synthesized core–shell structure. The results show that the monoaxially ordered PCL nanofiber-coated yarn had higher hydrophilicity due to the introduction of a layer of aligned PCL nanofiber shells onto the polyglycolic acid yarn core.

There are some property differences between highly ordered nanofibers and poorly ordered nanofibers. Cheng et al. [41] investigated the effect of matrix viscosity on the arrangement of multi-walled carbon nanotubes and the physical properties of high-density polyethylene/multi-walled carbon nanotube composite nanofibers prepared by co-extrusion of nanolayers. The results show that the highly ordered density polyethylene/multi-walled carbon nanotube composite nanofibers have higher decomposition temperatures and better mechanical properties. However, because three-dimensional (3D) networks are easily formed, poorly ordered nanofibers have higher electrical and thermal conductivity.

Nanofibers fabricated by electrospinning have been the ideal candidates for the energy and environment [42], wound healing [43,44], drug delivery [45,46], wastewater treatment [47], sensors [48] and air filtration [49] due to their high surface area-to-volume ratios. Especially, highly ordered nanofibers are very useful when they are used in applications such as field effect transistors [50], gas sensors [51], reinforced composite materials [52] and tissue engineering. Lin et al. [50] fabricated ordered nanofiber using poly(3-hexylthiophene)-graphene composite materials and produced field effect transistors. The results show that the geometry of ordered nanofibers can reduce the aggregation of graphene and thus improve carrier mobility. At the same time, the carrier mobility of field effect transistors made of ordered nanofibers is an order of magnitude higher than that of spin-coated-film field effect transistors. The mobility of ordered nanofibers’s field effect transistors is 1.82 cm^2^ V^−1^ s^−1^, and the mobility of spin-coated-film field effect transistors is 0.1 cm^2^ V^−1^ s^−1^. This result proves that ordered nanofibers can improve charge transfer efficiency and thus improve electrical properties in electronic devices such as field effect transistors. Wang et al. [51] spun highly ordered parallel nanofiber arrays of polyvinyl alcohol (PVA) by electrospinning and produced a gas sensor to detect NO_2_. The experimental results show that compared with the sensor made of disordered PVA nanofibers, the minimum detection concentration of ordered nanofibers is reduced by one-third. Meanwhile, the response and recovery time of the sensor with ordered nanofibers is 350 times and 130 times faster than those with disordered nanofibers, respectively. Ji et al. [52] prepared PAN composite nanofiber sheets with high orientation by electrospinning and hot stretching. The tensile strength and modulus of ordered PAN nanofiber sheets are 320.7% and 204.5% of those of disordered nanofiber sheets. Shao et al. [53] prepared novel scaffolds composed of multi-layer nanofiber fabrics by weaving nanofiber yarns with polylactic acid (PLA) and quercous serrata silk protein. The yarns were prepared by electrospinning. The results showed that the scaffolds had a high Young’s modulus and tensile strength, which could reach 417.65 MPa and 180.36 MPa, respectively, and were two to four times higher than those of non-woven fabrics under the same conditions. Therefore, the scaffold has a bionic structure, excellent mechanical properties and good biocompatibility, and has potential applications in bone tissue engineering.

**Figure 2:**
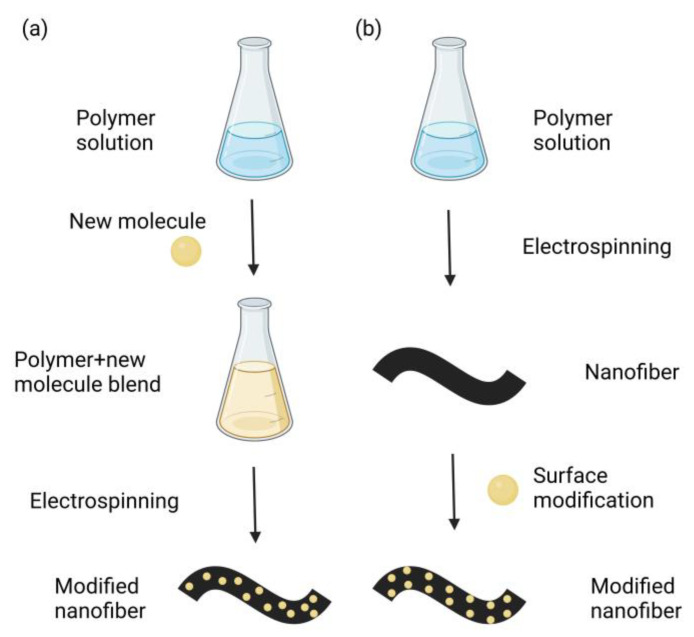
Two approaches to functionalizing nanofibers: (**a**) direct incorporation and (**b**) surface modification. Created with BioRender.com. Accessed on 10 April 2023.

Nanofibers can be easily functionalized to provide additional properties in various fields. Figure 2 shows two methods of nanofibers functionalization. The first method is direct incorporation. This method introduces the new molecule into the original solution to prepare a new blended solution. Then, the combined solution is used as a precursor to fabricate the modified nanofiber by electrospinning. The second method is surface modification. In this method, the nanofiber is spun by electrospinning first. Then, the new molecule is modified on the surface of the nanofiber to prepare modified nanofibers [54]. The direct incorporation method can make the new molecules more evenly distributed in the fiber, while the surface modification method only allows the new molecules to stay on the surface.

Various compounds can be used for nanofiber modification, such as polymers, nanoparticles, organic compounds and dyes, carbon nanomaterials and biomolecules [4]. Biomolecules have a larger surface area and porous structure than microscale materials do. Therefore, the capacity of biomolecule immobilization is increased, and biomolecules perform better than sensors [55]. For nanofiber scaffolds, proper surface functionalization improves intercellular adhesion and proliferation [56]. Functionalized synthetic scaffolds are effective at improving the physiological relevance of tissue-engineered grafts. Klabukov et al. [57] prepared flat microfibrous PCL scaffolds by emulsion electrospinning and introduced the pCMV-VEGF-165 plasmid into the microfibers. The experimental results showed that the functionalization of PCL by adding the pCMV-VEGF-165 plasmid improved vascularization within 33 days after implantation.

Each of the two methods of nanofiber functionalization has its advantages and disadvantages. The experimental procedure is more straightforward for the direct incorporation method, and the fibers are easier to prepare. The direct incorporation method enables the homogeneous blending of organic polymeric and functional materials. However, this method needs to overcome several factors in fabricating sensors, such as issues of aggregation and stability over the time/temperature/experimental process, quenching of fluorescence, and analyte diffusion into the nanofibers [4]. For the surface modification method, because a large surface area enhances the interaction with the analyte, leading to more sensitive detection, surface functionalization methods have better prospects for sensing applications. Additionally, the functionalized materials are distributed on the surface of the nanofibers. Nanofibers prepared by this method are able to achieve certain special functions, such as hydrophobicity [58].

Although some of the literature has summarized nanostructures for sensor-related applications, few articles specifically review highly ordered nanostructure arrays for sensors. Highly ordered nanofibers are widely used in the field of sensors because of their strong axial electron transport capacity and larger effective specific surface area. Here, we aim to summarize a series of ordered nanostructured arrays and their applications in various sensors. This review summarizes the current progress in electrospinning for fabricating highly ordered nanofibers. The multiple electrospinning technologies and their principles of forming highly ordered nanostructures are reviewed first, and then the sensor applications of highly ordered nanofibers are introduced.

## 2. Electrospinning Methods for Forming Highly Ordered Nanofibers

**Figure 3:**
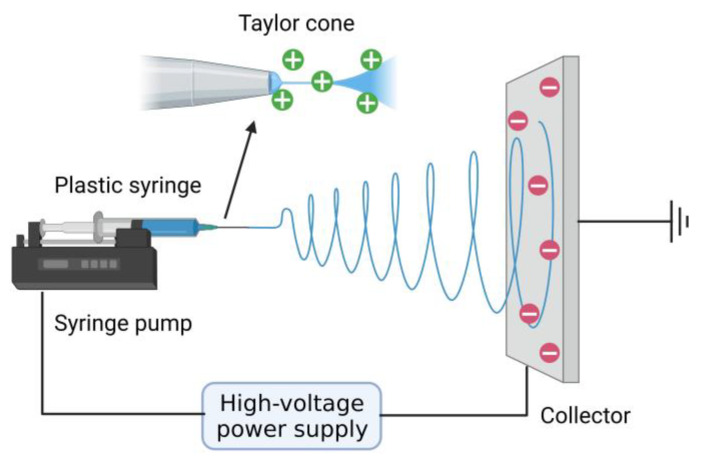
Schematic diagram of typical electrospinning devices used to fabricate normal nanofibers. Created with BioRender.com. Accessed on 10 April 2023.

Figure 3 shows the schematic diagram of typical electrospinning devices used to fabricate normal nanofibers. A typical electrospinning device includes three units: a high-voltage power supply, a propulsion unit, and a receiving unit. Among them, the propulsion unit includes a plastic syringe and syringe pump. The receiving unit is usually a fixed metal plate or rotating shaft [59]. The syringe is filled with a polymer solution or polymer melt. When a static voltage of a dozen or even tens of kilovolts is applied to the needle connected to the positive terminal and the collector connected to the negative terminal, a conical droplet is formed at the needle. The conical droplet is called a “Taylor cone”. When the applied voltage exceeds a critical value, the electrostatic field force on the polymer solution becomes greater than the surface tension of the solution. The charged jet is then ejected from the Taylor cone and collected on the negatively charged collector. The volatilization of the polymer solvent and the solidification of the polymer melt are the leading causes of instability in polymer jets. The charged jet is ejected in very fine fibers by the force of the electric field. Jets move in a spiral and are deposited on the surface of the collector to obtain nanofibers [55].

Many parameters need to be paid attention to in the process of electrospinning which will affect the quality of nanofibers—for example, polymer molecular weight and solution concentration, applied voltage, spinning distance and external environment. Changes in temperature and humidity in the external environment are also significant. After spinning, nanofibers are characterized in many ways. Their morphology is characterized by X-ray diffraction (XRD), scanning electron microscopy (SEM), and transmission electron microscopy (TEM). XRD is mainly used to test the degree of crystallization of the sample. SEM is used to obtain the microscopic morphology of nanofibers, and TEM is used to observe the microscopic morphology with a high resolution. Performance characterization is mainly a test of mechanical properties and electrical properties.

The approach to designing highly ordered nanofiber materials is to introduce external forces to counteract the bending jet’s instability. Highly ordered nanofibers can be easily fabricated by improved electrospinning by introducing external forces, such as magnetic [60], electric [23,61], and mechanical forces [62,63]. Additionally, some parameters in the electrostatic spinning process can also affect the alignment of the fiber. Paskiabi et al. [64] quantitatively determined the effect of four electrostatic spinning parameters on PCL/polycaprolactone nanofiber alignment by the artificial neural network. The results showed that concentration and working distance have little effect on fiber alignment, while applied voltage and drum speed are the critical parameters for nanofiber alignment. More highly ordered nanofibers will be obtained when the applied voltage or the drum speed is increased. However, it is worth noting that to obtain the best nanofibers, as long as one of the parameters is at its maximum value, the other should not be too large. It has also been found that as the drum speed increases, the nanofiber’s alignment increases while its average diameter decreases [65].

### 2.1. Directing Writing Melt Electrospinning with Controllable Platform

Currently, most of the polymer fibers produced by melt electrospinning technology exist in the form of disorderly non-woven fabrics, which limits the application of electrospinning technology in ordered structures. Li et al. [66] reviewed the application of electrospinning technology in tissue engineering scaffolds and proposed that using electrospinning and rapid prototyping technology to obtain tissue engineering scaffolds with optimized orientation is a significant development direction of electrospinning technology. Liu et al. [67] introduced the controllable forming platform to electrospinning technology and explored the influence of process parameters such as nozzle moving speed, spinning voltage and receiving distance on fiber deposition characteristics using a direct writing melt electrospinning molding device. Still, the specific mechanism of action needed to be studied more in-depth.

**Figure 4:**
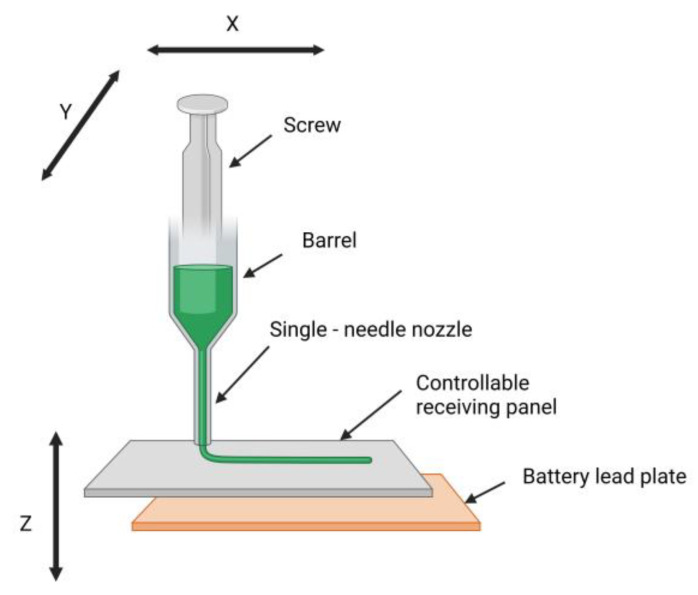
Schematic diagram of directing writing melt electrospinning with the controllable platform. Created with BioRender.com. Accessed on 10 April 2023.

Chen et al. combined melt electrospinning technology with a 3D motion platform and used a self-designed melt electrospinning controlled-forming experimental device to conduct the direct writing melt electrospinning of PCL and obtained highly ordered nanofibers. Figure 4 shows the schematic diagram of directing writing melt electrospinning with the controllable platform, which mainly includes a screw, barrel, single-needle nozzle, controllable receiving panel and battery lead plate. The motion control system uses mechanical force to control the movement of the single-needle nozzle in the X–Y plane. The receiving plate can be raised and dropped to control the Z direction, which can quickly realize the control of the spinning distance and the molding of the 3D-ordered structure. Conventional melt electrospinning techniques are challenging to use to form highly ordered fibers. However, highly ordered nanofibers can be easily fabricated by combining melt electrospinning technology with a 3D motion platform [68].

**Figure 5:**
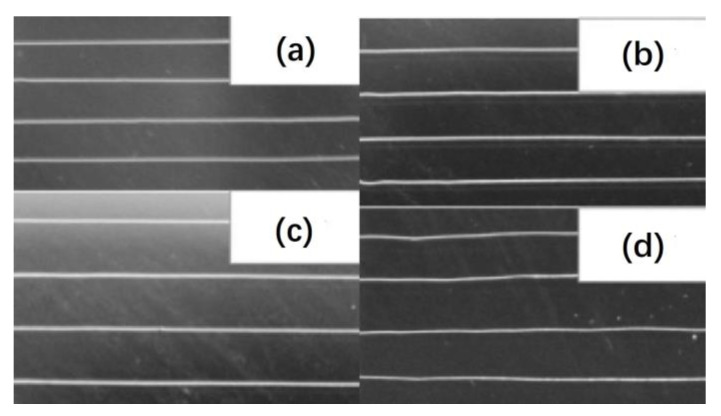
SEM images of highly ordered PCL fibers fabricated by directing writing melt electrospinning with a controllable platform at different voltages and moving speeds: (**a**) 15 kV, 500 mm/min; (**b**) 20 kV, 700 mm/min; (**c**) 25 kV, 900 mm/min; (**d**) 30 kV, 1200 mm/min. Reproduced with permission from ref. [69].

Figure 5 shows SEM images of highly ordered PCL fibers fabricated by directing writing melt electrospinning with a controllable platform at different voltages and moving speeds. In attempting to focus the deposition of the electrospun nanofibers to obtain highly ordered nanofibers, a general study has been conducted [69]. When electrospinning is used in the direct writing mode, the polymer jet does not undergo significant buckling instabilities. At the same time, the nanofiber can be drawn by the controllable platform or spinneret to obtain finer fibers.

**Figure 6:**
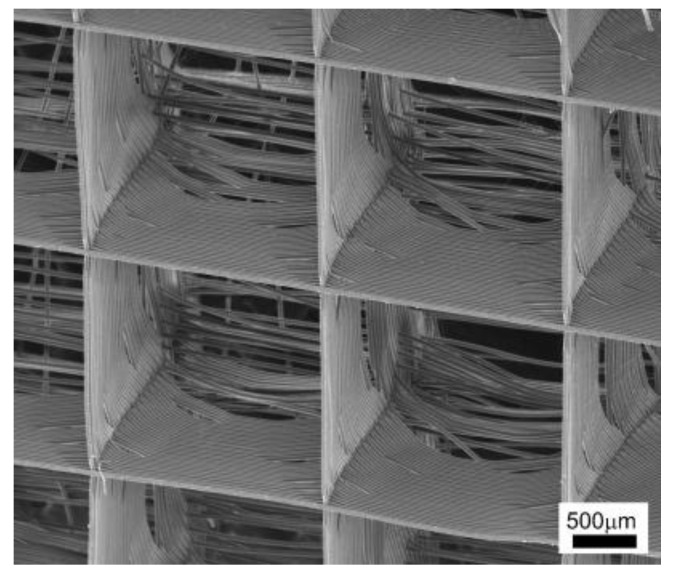
SEM images of melt electrospun PCL scaffold with highly ordered microstructures. Reprinted from ref. [70] with permission from WILEY-VCH Verlag GmbH & Co. KGaA, Weinheim, copyright 2011.

Figure 6 shows the SEM images of a melt electrospun PCL scaffold with highly ordered microstructures [70]. It indicates that melt electrospinning with a controllable platform or spinneret has excellent potential to fabricate 3D-ordered and other-ordered microstructures.

**Figure 7:**
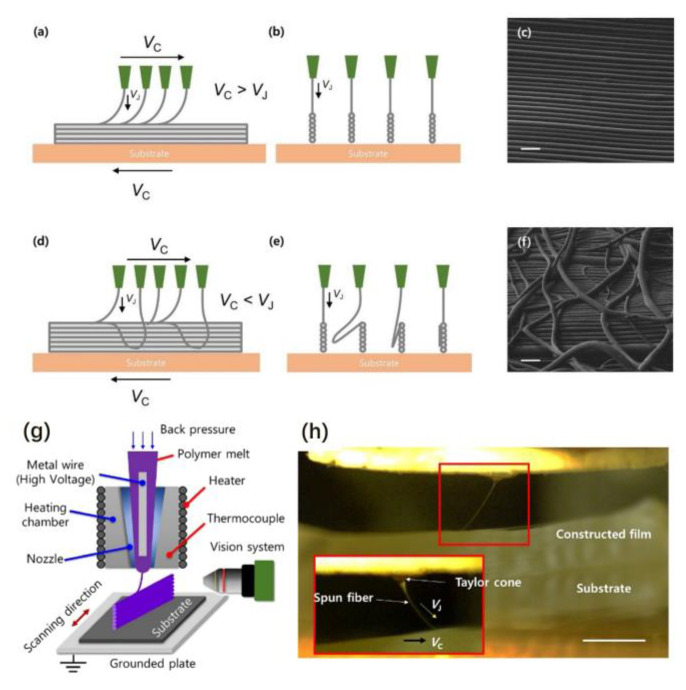
Different structures of the spun fiber under different velocities: (**a**) Schematic diagram of the spun fiber when V_C_ is faster than V_J_. (**b**) Lateral view of (**a**). (**c**) Magnified image of (**a**). (**d**) Schematic diagram of the spun fiber when V_J_ is faster than V_C_. (**e**) Lateral view of (**d**). (**f**) Magnified image of (**d**). (**g**) Schematic image of melt electrospinning device. (**h**) In situ image of electrospinning process. Scale bars are 50 μm in (**c**,**f**), and 10 mm in (**h**). Reprinted from ref. [71] with permission from IOP Publishing Ltd., copyright 2018.

Lee et al. built and used the melt electrospinning system to fabricate highly ordered nanofibers. Figure 7g shows the schematic image of the melt electrospinning setup. The melt electrospinning system comprises a metal wire with a high voltage, a heating chamber, nozzle, heater, thermocouple vision system and substrate. Figure 7h shows the in situ image of the electrospinning process. The constructed film is a narrow vertical wall fabricated by polyether block amide melting. The Taylor cone formed by the tip of the spun fiber can be seen clearly from the magnified image. There are two velocities in the picture; one is the velocity of the jet (V_J_), and another is the velocity of the collecting substrate (Vc). Different microstructures can be obtained by adjusting the speed of the two velocities [71].

The different structures of the spun fiber under different velocities are shown in Figure 7. To obtain highly ordered nanofiber, V_C_ should be faster than V_J_. When V_J_ is faster than V_C_, the redundant polymer will deposit the narrow vertical wall, and it will cause a disordered structure.

It has been found that for direct writing melt electrospinning technology, the effect of increasing voltage on fiber diameter is not apparent due to the short receiving distance and the short action time of the electric field in fiber elongation refining. In the direct writing melt electrospinning process, the matching nozzle’s moving velocity and jet falling velocity are the key to the orderly deposition of fibers. With the increase in the receiving distance, the whipping section of the jet is introduced, and the fiber order worsens. The fiber deposition morphology is the best when the receiving distance is 3 mm. The falling velocity of the jet increases with an increase in voltage. However, when the voltage is too high, the jet whips too much under the action of the electric field force, and the ordered fiber cannot be obtained.

### 2.2. Magnetic-Field-Assisted Electrospinning

Electrospinning itself is an uncontrollable technology. Due to the influence of various factors in the spinning process, jet whipping is intense and unstable, so the nanofibers prepared by ordinary electrospinning are mostly randomly arranged non-woven fibers. Magnetic field-assisted electrospinning has attracted much attention because of its ability to produce large-area-oriented fibers.

**Figure 8:**
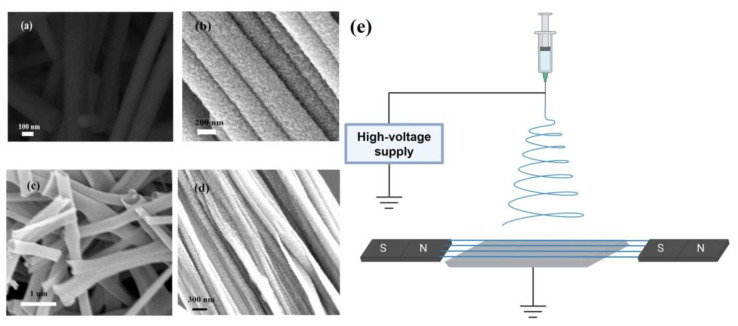
SEM images of (**a**) disordered TiO_2_ nanofibers, (**b**) ordered TiO_2_ nanofibers, (**c**) disordered TiO_2_ nanotubes and (**d**) ordered TiO_2_ nanotubes. Reprinted from ref. [60] with permission from Elsevier Ltd. and Techna Group S.r.l., copyright 2019. (**e**) Schematic diagram of magnetic-field-assisted electrospinning device for forming highly ordered nanofibers. Created with BioRender.com. Accessed on 10 April 2023.

Wang et al. [60] used magnetic field-assisted electrospinning to prepare TiO_2_ nanofibers/nanotubes, ordered semiconductor materials, and studied their photocatalytic performance. Due to the introduction of the magnetic field, the band gap of TiO_2_ nanomaterials was reduced, and the contact with organic pollutants was more adequate, thus improving photocatalytic performance. The schematic diagram of a magnetic field-assisted electrospinning device for forming highly ordered nanofibers is shown in Figure 8e. This method uses a magnet’s polarity to control the electric field’s distribution in an electrospinning system. The experimental results show that the bending instability of the charged liquid jet can be effectively controlled by placing magnets on both sides of the fiber formation path, thus producing highly ordered nanofibers.

The prepared nanofibers of various structures were observed using SEM. In Figure 8a,c, the nanofibers and the nanotubes look disordered and smooth. The samples are highly ordered in Figure 8b,d because of the addition of a magnetic field. The highly ordered nanomaterials have a fewer smooth surfaces and longer lengths than the disordered nanomaterials do. These particular surface morphologies can provide more active sites. According to the above results, it can be concluded that the technique of preparing ordered TiO_2_ nanomaterials by magnetic-field-assisted electrospinning is mature and is expected to be extended to other materials.

### 2.3. Electrospinning with Parallel Electrode Method

The two main ways to obtain highly ordered nanofibers are used to improve the collection apparatus. One way is to use a rotating collector, which is grounded [51,52], and another is to use the parallel electrode method [72,73,74,75]. The parallel electrode method fabricates ordered nanofibers using two stationary and separated conducting electrodes. The parallelly placed auxiliary electrodes can generate a symmetrical auxiliary electric field that can dampen the bending instability of the electrospinning jet perpendicular to the direction of alignment to enable aligned deposition. When the nanofibers fall, they are subjected to an electric force from the parallel electrodes. Additionally, then, they are collected between the two electrodes. Nevertheless, the degree of order of the nanofibers decreases with an increase in the spinning time in the parallel electrode method.

**Figure 9:**
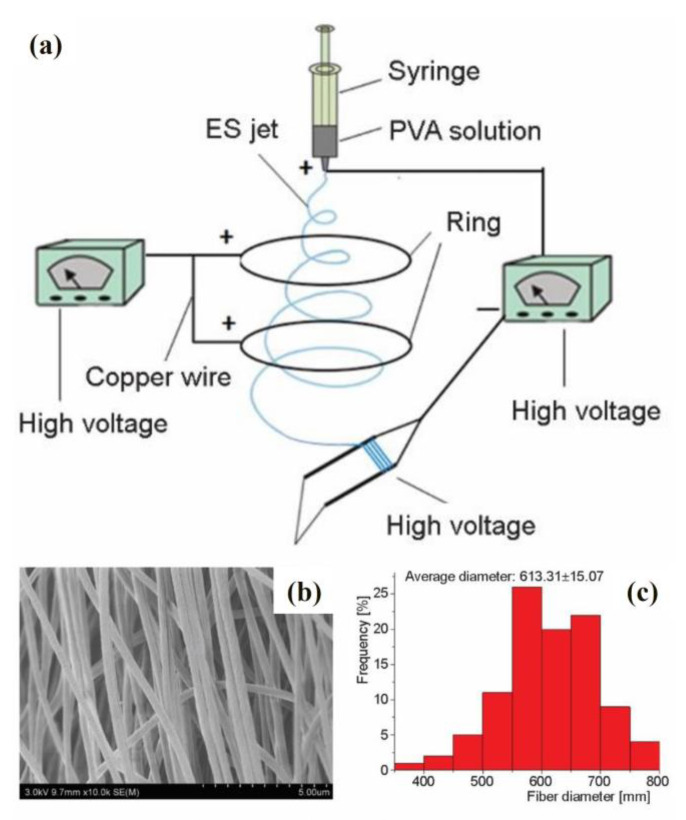
(**a**) Schematic diagram of improved parallel electrode method device for forming highly ordered nanofibers. SEM image (**b**) and diameter distribution (**c**) of the highly ordered nanofibers with the 6 cm distance between two copper rings. Reproduced with permission from ref. [61].

An improved parallel electrode method was reported to solve this problem. This method is based on the original device but adds two parallel copper rings between the parallel electrodes and the needles. These two copper rings are positively charged and connected to the high-voltage device. The new method improves the diameter distribution of the nanofibers and increases their highest degree of order. The schematic diagram of this improved parallel electrode method device for forming highly ordered nanofibers is shown in Figure 9a. The device contains one syringe with one needle, two copper rings, two high-voltage generators and one parallel-electrode collector. The right high-voltage generator in Figure 9a is an applied-voltage generator. The positive electrode of the applied-voltage generator is connected to the needle, and the negative electrode is connected to the parallel-electrode collector. The high-voltage generator on the left in Figure 9a is a ring-voltage generator. The positive electrode of the ring-voltage generator is connected to two copper rings through the copper wire [61].

They adjusted the distance from 2 to 8 cm between the two copper rings to obtain the highest degree of order for the nanofibers. The SEM image (Figure 9b) and diameter distribution (Figure 9c) of the highly ordered nanofibers are provided when the distance between two copper rings is 6 cm. In this condition, the nanofiber has the most uniform diameter distribution and highest degree of order, which could be up to 82%. The results show that this improved parallel electrode method can produce highly ordered electrospun nanofibers.

**Figure 10:**
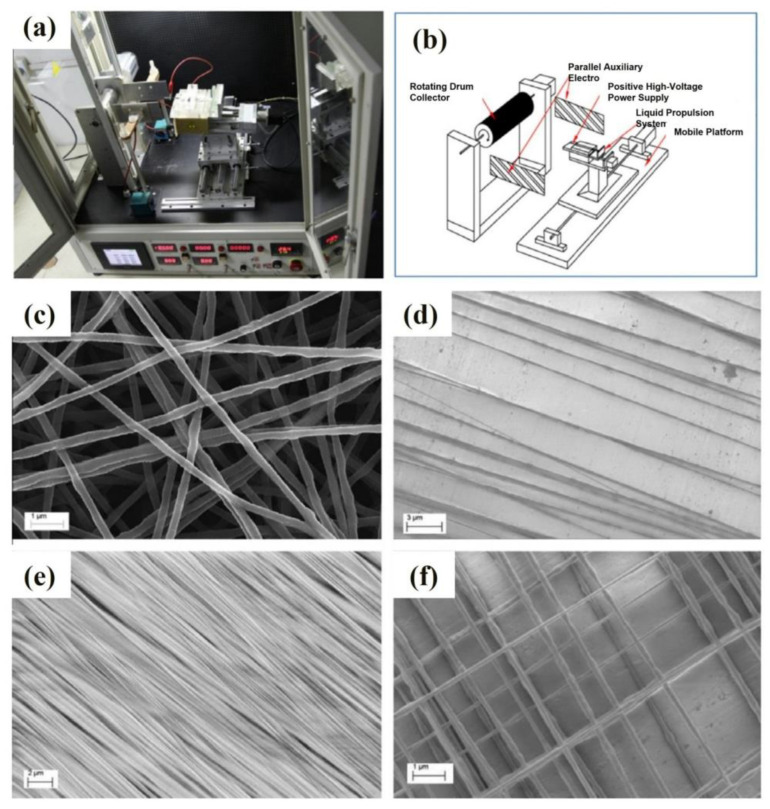
Photograph (**a**) and schematic diagram (**b**) of the electrospinning setup with parallel electrodes. SEM images of polyethylene oxide-electrospun nanofibers with different microstructures (**c**) disordered nanofiber, (**d**) uniaxially ordered nanofiber with low density, (**e**) uniaxially ordered nanofiber with high density, and (**f**) biaxially ordered nanofiber. Reprinted from ref. [23] with permission from Elsevier Ltd., copyright 2012.

Su et al. [23] improved the traditional electrospinning setup to obtain highly ordered nanofibers. Figure 10a,b shows the photograph and schematic diagram of the electrospinning setup with parallel electrodes, respectively. They added a pair of parallel auxiliary electrodes and improved the speed of the rotating drum collector. In traditional electrospinning, the polymer jet usually exhibits bending instabilities because of the repulsive forces between the charges carried within the jet. Therefore, the nanofiber fabricated by traditional electrospinning is mostly disordered and has limited applications.

Disordered nanofibers fabricated by an improved electrospinning setup are shown in Figure 10c. Uniaxially ordered nanofibers with different densities and biaxially ordered nanofibers with different hole sizes can be fabricated by adjusting parameters, such as spinning time, the movement speed and the distance of the platform. Uniaxially ordered fibers with different fiber densities are shown in Figure 10d,e. Figure 10f shows a biaxially ordered nanofiber with a hole size of 1 μm. Mechanical and electrical property tests were conducted to explore the impact of different structures of nanofibers on their properties. Tensile tests were conducted on the nanofibers under different load levels. Their tensile strength and modulus are obtained by analyzing their stress–strain curves. The results show that the strength of biaxially ordered nanofibers is much higher than that of disordered nanofibers but slightly lower than that of uniaxial ordered nanofibers. The orderly arrangement of nanofibers is conducive to forming conductive paths. Uniaxially ordered nanofibers have the highest electrical conductivity, followed by biaxially ordered nanofibers, and finally by disordered nanofibers.

### 2.4. Electrospinning with Parallel Double Thin Plate Collector

**Figure 11:**
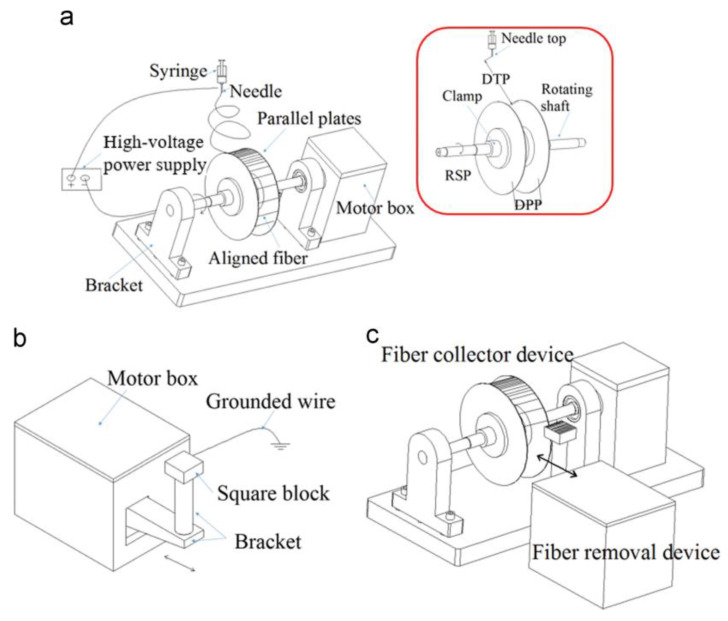
Schematic drawing of (**a**) fiber-collecting equipment; (**b**) removal equipment; and (**c**) all components of the electrospinning equipment. Reprinted from ref. [76] with permission from Elsevier BV, copyright 2015.

Conventional electrospinning can be used to easily fabricate 2D-ordered nanofibrous structures with finite thickness, but 3D-ordered fibrous scaffolds cannot. Mi et al. developed a novel electrospinning device to generate 3D-electrospun fibrous scaffolds with highly ordered nanofibers and controllable thickness. Figure 11a shows the schematic drawing of the fiber-collecting equipment. The fiber-collecti ng equipment contains a syringe with a needle, two parallel plates, a high-voltage power supply, a motor box and a bracket. The clamps fixed two parallel plates to the rotating shaft, and the fibers were collected between the two thin plates. Figure 11b shows the schematic drawing of the fiber removal equipment. The fiber removal equipment includes a motor box, grounded wire, square block and bracket. The motor box supplied power for the square block to move back and forth between the two parallel plates at a fixed speed. All components of the equipment, including the fiber-collecting and removal equipment, are shown in Figure 11c. In this condition, highly ordered nanofibers can be quickly and completely removed and put into the square block [76].

**Figure 12:**
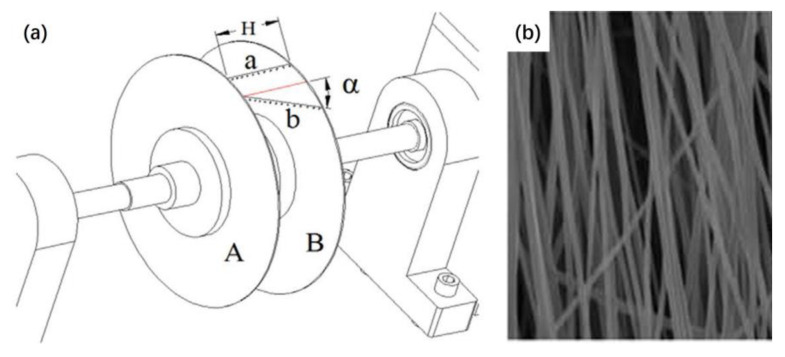
(**a**) Possible distribution of nanofibers in fiber-collecting equipment. (**b**) SEM image of highly ordered PVA nanofibers. Reprinted from ref. [76] with permission from Elsevier BV, copyright 2015.

Figure 12a shows the possible distribution of nanofibers in the fiber-collecting equipment. The fibers perpendicular to two parallel plates are ordered fibers, and they were named a. Other randomly aligned fibers are disordered fibers, and they were named b. The angle between a and b is α. The fiber alignment degree was analyzed through the degree of deviation of the fibers from the standard angle. Figure 12b shows the SEM image of highly ordered PVA nanofibers. The sample in the picture has the best alignment degree, 76% of the fibers in the picture are oriented within approximately ±5%, and their diameter is 224 ± 50 nm. The results indicate that this electrospinning equipment is a powerful tool for fabricating controllable 3D structures.

### 2.5. Electrospinning with Conductive Substrate Collector

**Figure 13:**
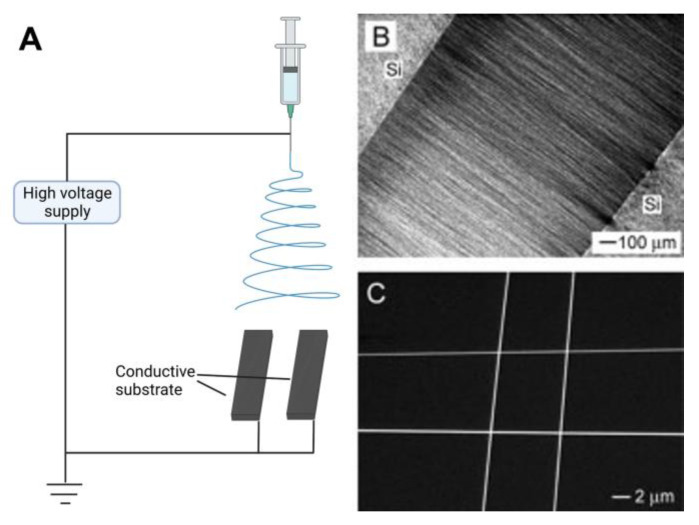
(**A**) Schematic diagram of the electrospinning device with two conductive substrates. Created with BioRender.com. Accessed on 10 April 2023. (**B**) Optical micrograph of highly ordered poly(vinylpyrrolidone) nanofibers fabricated by the electrospinning device. (**C**) SEM image of a 2 × 2 array of poly(vinylpyrrolidone) nanofibers crossbar junctions. Reprinted from ref. [77] with permission from WILEY-VCH Verlag GmbH & Co. KGaA, Weinheim, copyright 2004.

Because of the bending instability of the high-charge electric jet, the electrospun fibers are deposited on the surface of the collector (conductive substrate) in the form of disordered chaos. For a long time, it has been hard to collect these highly ordered nanofibers directly. Li et al. [77] invented a method that fabricates highly ordered nanofibers and facilitates deposition or transfers to a solid substrate surface for device fabrication. The schematic diagram of the electrospinning device is shown in Figure 13A. The device consists of a power supply, a needle and a collector. The collector comprises two conductive silicon strips with a void gap between them. By adjusting the width of this gap, various nanofibers with different lengths from hundreds of micrometers to several centimeters can be obtained. Figure 13B shows the optical micrograph of highly ordered poly(vinylpyrrolidone) nanofibers fabricated by this electrospinning device. As seen in Figure 13B, the fibers are orderly arranged in the gap, and their direction is perpendicular to the substrate. In contrast, the fibers deposited on the substrate surface are disordered. After electrospinning, the nanofibers can be conveniently transferred to another substrate surface by vertically moving the substrate. By moving the collector, the position and direction of the intermediate fibers can be easily controlled. In this way, the fibers can be spun into a multi-layer structure layer by layer, with different fibers having different orientations. 

Figure 13C shows an SEM image of a 2 × 2 array of crossbar junctions of poly(vinylpyrrolidone) nanofibers. By adjusting the collection time, the density between each layer of nanofibers can be more or less controlled. At the same time, people can deposit a nanofiber in the gap and then transfer it to the patterned electrode to obtain single fiber-based equipment and manufacturing. Compared with other methods, this technology has the advantages of simplicity, versatility and directness [77].

**Figure 14:**
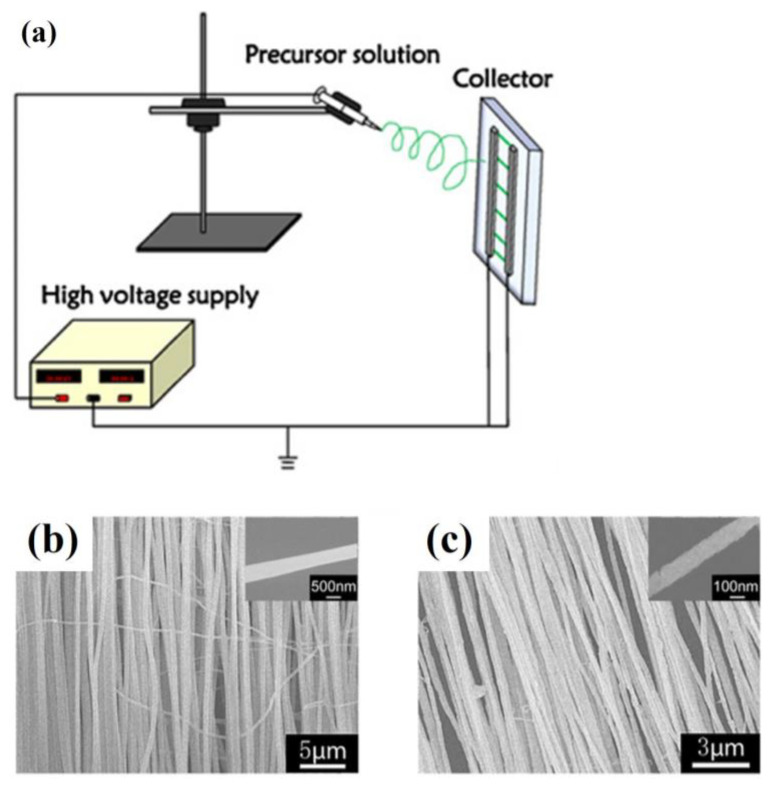
(**a**) The schematic image of the electrospinning device for one humidity sensor. SEM diagrams of highly ordered Ba_χ_Sr_1−χ_TiO_3_ nanofibers (**b**) before and (**c**) after annealing at 800 °C for 2 h. Reprinted from ref. [30] with permission from Elsevier BV, copyright 2012.

Highly ordered Ba_0.8_Sr_0.2_TiO_3_ nanofibers were fabricated by special electrospinning with calcination. Figure 14a shows the schematic image of the electrospinning device for one humidity sensor. The unique feature of this device is the collector. The collector is assembled by mounting two aluminum electrodes on an insulating substrate. Figure 14b shows SEM diagrams of highly ordered Ba_χ_Sr_1−χ_TiO_3_ nanofibers before calcination at 800 °C. Before calcination, the nanofiber is smooth and united of a diameter between 300~500 nm. Figure 14c shows SEM diagrams of highly ordered Ba_χ_Sr_1−χ_TiO_3_ nanofibers after calcination at 800 °C. After calcination, the surface of the nanofiber became rough, and the average diameter of the Ba_χ_Sr_1−χ_TiO_3_ nanofibers reduced to about 100 nm. This shrinkage arises from the evaporation of volatile organic compounds that form Ba_0.8_Sr_0.2_TiO_3_ nanofibers at high temperatures [30].

This new electrospinning method is improved by separating the traditional collector electrodes. It enables the simple generation of uniaxially oriented nanofibers with various compositions and properties. Additionally, it is possible to stack aligned nanofibers into multi-layer films with a well-defined hierarchical structure. This novel electrospinning technique will have potential applications in fabricating nanofiber structures, devices, and systems.

### 2.6. Electrospinning with Rotating Drums

Various electrospinning collectors, such as rotating drums, wheels, or parallel plates, are used to obtain the desired nanofiber orientations. Electrospinning with a rotating disc that can increase the tensile properties of a single electrospun nanofiber has been reported [78].

**Figure 15:**
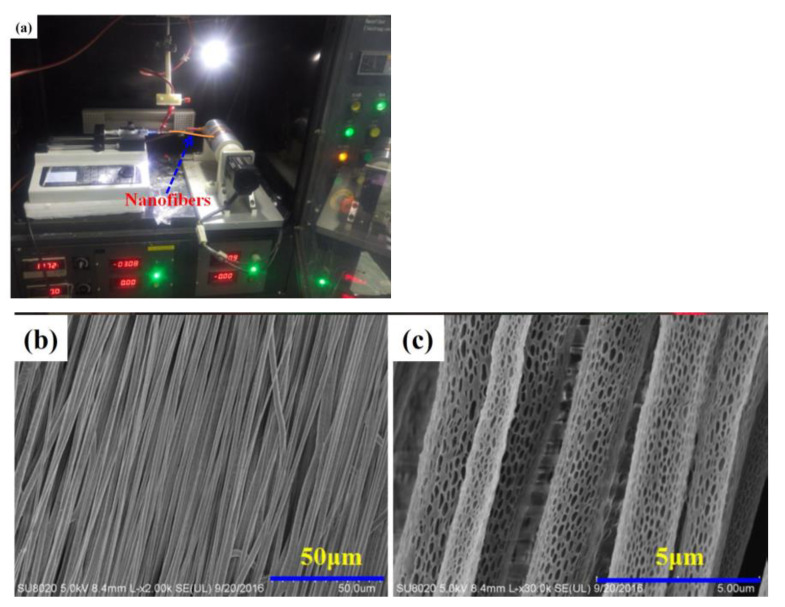
(**a**) Images of the electrospinning device for highly ordered piezoelectric PLLA nanofibers. SEM images of highly ordered PLLA nanofibers (**b**) 50 μm and (**c**) 5 μm in diameter. Reprinted from ref. [62] with permission from Springer Science Business Media New York, copyright 2017.

Zhu et al. first demonstrated a method for fabricating permanently polar L-polylactic acid (PLLA) polymer nanofibers on a comb electrode along the ordered nanofiber axis. This method is achieved in one step. Figure 15a shows the images of the electrospinning device for highly ordered piezoelectric PLLA nanofibers. The electrospun solution comprised 0.3 g of PLLA powder and 3 mL of a dichloromethane solvent. The prepared comb electrodes were fixed on the rotating collector to collect PLLA nanofibers. The disk rotates at 1800 revolutions per minute (rpm). The nozzle and collector are 10 cm apart. The applied force comes from injecting a high direct electric field into the PLLA nanofiber polymer solution. The entire dipole component along the main carbon chain can be arranged by electrospinning along the nanofiber direction. Figure 15b,c shows the SEM images of highly ordered PLLA nanofibers at different magnifications. The nanofibers range between 100 nm and 2 μm in diameter [62].

Electrospun nanofibers’ mechanical properties are determined by the polymer’s molecular orientation and the fibers’ physical orientation. Polymer jets under the effect of an electrostatic field have high molecular orientation due to the high elongation strain and shear force [36]. As a result, electrospun nanofibers have higher strength than conventional fibers do. Additionally, the physical orientation of electrospun fibers affects the fiber strength [79]. In the physical orientation direction, the mechanical strength of nanofibers is higher. 

Electrospinning with rotating drums is a relatively easy way to obtain ordered nanofibers. However, to obtain more highly aligned nanofibers, the speed of the drum needs to be appropriate. The drum surface’s linear velocity should match the solvent’s evaporation rate so that the fibers are deposited and adsorbed on the drum surface. Randomly oriented fibers are obtained on the drum at a speed that is less than the fiber pickup speed. In contrast, the fiber pickup speed can cause the fibers to break at higher speeds, and continuous fibers cannot be collected.

### 2.7. Conjugate Electrospinning

**Figure 16:**
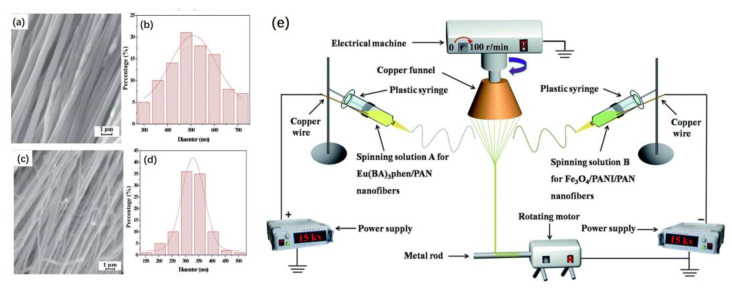
(**a**) SEM image of heterogeneous [Fe_3_O_4_/PANI/PAN]//[Eu (BA)3phen/PAN] nanofibers. (**b**) Diameter distribution histogram of heterogeneous [Fe_3_O_4_/PANI/PAN]//[Eu (BA)3phen/PAN] nanofibers. (**c**) SEM image of homogeneous [Fe_3_O_4_/PANI/Eu (BA)3phen/PAN] nanofibers. (**d**) Diameter distribution histogram of homogeneous [Fe_3_O_4_/PANI/Eu (BA)3phen/PAN] nanofibers. (**e**) Schematic drawing of the conjugate electrospinning equipment. Reproduced with permission from ref. [63].

Fan et al. successfully prepared one-dimensional heterogeneous nanofibers with tri-functionality of fluorescence, superparamagnetism and electrical conductivity by the conjugate electrospinning technique. The fibers were made up of polyaniline (PANI), PAN, benzoic acid (BA) and phen(1,10-phenanthroline). The results show that heterogeneous nanofiber yarns possess a larger length-to-diameter ratio and a more uniform diameter than the homogeneous nanofiber yarns do. At the same time, the nanofibers in heterogeneous yarns have a higher degree of orientation. Figure 16e shows a schematic drawing of the conjugate electrospinning equipment. Spinning solution A and spinning solution B are injected into two syringes fitted with spinnerets. The left spinneret is connected to a positively charged high-voltage power supply with a copper wire. In contrast, the front spinneret on the right is connected to a negatively charged high-voltage power supply with a copper wire. Two plastic syringes are placed symmetrically on either side of the electrical machine. The copper funnel is connected to a grounded electrical machine that can control the rotation speed. The applied voltage is adjusted to ±15 kV or so. The distance from the spinneret’s top to the copper funnel’s side is 10 cm. The collection device, located beneath a copper funnel, consists of a rotating motor and a metal rod. The lower edge of the funnel should be 18 cm away from the collecting device. PAN-based continuous nanofibers were fabricated at a relative humidity of 20 ± 5% and a temperature of 20 ± 5 °C [63].

Figure 16a, b shows the SEM image and diameter distribution histogram of heterogeneous [Fe_3_O_4_/PANI/PAN]//[Eu(BA)_3_phen/PAN] nanofibers, respectively. As seen in Figure 16a, under high-magnification SEM, the nanofibers have a high degree of orientation and some aggregation of Fe_3_O_4_ nanoparticles on the surface. As seen in Figure 16b, the average diameter of the nanofibers is 508 ± 8 nm. Figure 16c,d shows the SEM image and diameter distribution histogram of homogeneous [Fe_3_O_4_/PANI/Eu(BA)_3_phen/PAN] nanofibers, respectively. As seen in Figure 16c, the homogeneous nanofibers also have a high degree of orientation and the Fe_3_O_4_ nanoparticles are distributed on each nanofiber. As seen in Figure 16d, the average diameter of the nanofibers is 329 ± 2 nm. 

In summary, the conjugate electrospinning method can successfully produce heterogeneous nanofibers. Although the heterogeneous nanofiber yarns are twisted, the nanofiber yarns possess a high degree of alignment and orientation. Additionally, nanofiber yarns have great potential in the field of gas sensors.

### 2.8. Other Electrospinning Methods

**Figure 17:**
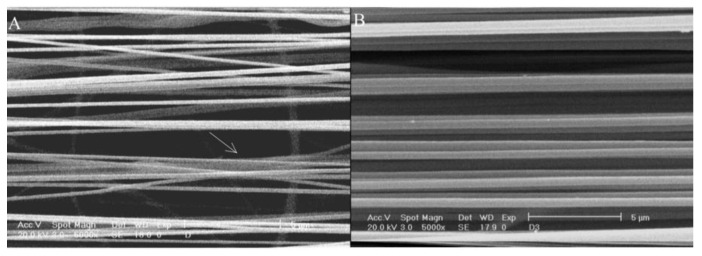
SEM images of highly ordered PAN nanofiber bundles (**A**) of a diameter of 240 nm and (**B**) of a diameter of 470 nm. Reprinted from ref. [80] with permission from Wiley Periodicals, Inc., copyright 2008.

In addition to improving the collection device, highly ordered nanofibers can be obtained by a modified method of preparing bundles such as using multi-filament yarn. Esrafilzadeh et al. studied the crystallization sequence and mechanical properties of PAN nanofibers by electrospinning and post-processing. To maintain the tension of the nanofibers during post-processing, an improved method of preparing nanofiber bundles, such as by using multi-filament yarn, was used to successfully control the arrangement and linear density of nanofiber bundles. With the increase in the diameter of nanofibers, the orientation of nanofibers improves obviously. Figure 17A shows the SEM images of highly ordered PAN nanofiber bundles of a diameter of 240 nm, and Figure 17B shows those of a diameter of 470 nm. The figure shows some tangles in the nanofibers with fine diameters but not in the nanofibers with coarse diameters [80].

Li et al. [81] prepared a cellulose nanocrystal–poly(lactic acid) nanocomposite random fiber mat and ordered-fiber yarn by emulsion electrospinning. They prepared disordered and ordered fiber mats with drum and disk electrospinning devices. The purpose of emulsion electrospinning is to prepare nanofibers with core–shell structure. The nanofibers prepared by this method can improve the encapsulation efficiency of bioactive compounds to achieve targeted drug delivery.

Electrospinning technology contains both far-field electrospinning and near-field electrospinning abilities. Near-field electrospinning is also called near-field direct writing technology or electrofluidic printing technology. The near-field direct-write electrospinning process works on the principle that highly ordered nanofibers can be prepared with the aid of precision motion control when the spacing between the collection device and the nozzle is controlled in the range from 0.5 to 5 mm [82]. Mai et al. [83] prepared PCL/CO/UA composite fibers with highly ordered structures by 3D near-field electrospinning. Cell proliferation experiments showed that both the PCL/CO/UA composite fibers and the post-crosslinking PCL/CO/UA composite fibers were non-cytotoxic and promoted cell proliferation and adhesion. This indicates the potential application of PCL/CO/UA composite fibers fabricated by near-field electrospinning in biomedical settings.

## 3. Sensor Application of Highly Ordered Electrospinning Nanofibers

Generally, the receptor and the signal transducer form the sensor are indispensable. Both are instrumental in contributing to the ultimate performance of the sensor and in broadening its field of application. There are two major performance indicators for the sensors: detection performance and signal conduction performance. Both are influenced by the performance of the nano additives in the system. The detection performance of the sensor is also referred to as receptor performance. The key performance metrics of highly ordered nanofibers are different for different sensor applications. Generally speaking, the degree of order of a nanofiber affects the electron transfer efficiency and thus determines the sensor’s performance. At the same time, the addition of different materials to the fiber directly affects its electrochemical performance. In some special cases, such as in pressure sensors, the diameter of the fiber also affects the sensitivity of the sensor [71]. An excellent sensor should have high selectivity, a low detection limit and a short response time. An electrochemical sensor with excellent performance should satisfy the following three points. First, it should have high selectivity for detectors, i.e., it should be able to accurately distinguish detectors from non-detectors. Second, an excellent electrochemical sensor should have low detection limits. We want it to be able to detect even low levels of detectors. Finally, a good sensor should be able to obtain the detection result as soon as possible, i.e., it should have a short response time [55].

### 3.1. Pressure Sensor

Melt electrospinning was reported to produce a thin film made of polyether block amide. A thin film for resistive pressure sensors can be obtained by arranging the spun cylindrical fibers in an orderly manner into a narrow vertical wall. The polystyrene sulfonate 3,4-ethylene dioxythiophenetrodes in the film are coated with poly(3,4-ethylene dioxythiophene) polystyrene sulfonate. When pressure is applied to the sensor, the contact area between the electrode and the film changes, this change produces a sensitive current response, and a range of pressure changes can be detected [71].

**Figure 18:**
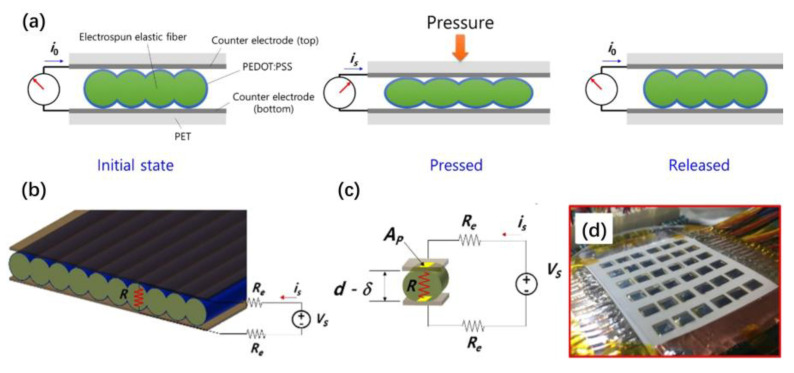
SEM images of highly ordered PAN nanofiber bundles (**a**) Schematic drawing of the working principle of the pressure sensor. (**b**)The schematic diagram of the simplified pressure sensor model. (**c**) The schematic diagram of an equivalent electrical circuit for the sensor. (**d**) The multi-touch interface with 36 pixels of the pressure sensor. Reprinted from ref. [71] with permission from IOP Publishing Ltd., copyright 2018.

Figure 18a shows a schematic drawing of the working principle of the pressure sensor. The green part represents the periodically ordered electrospun elastic fibers, the blue part represents the poly (3,4-ethylene dioxythiophene) polystyrene sulfonate-coated conducting layer, and the dark gray part represents the counter electrodes. Figure 18b shows the simplified pressure sensor model, and Figure 18c shows the schematic diagram of an equivalent electrical circuit for the sensor. They assume that coated resistive thin film behaves like an Ohmic conductor, and that the contact area affects its electrical resistance. The reduced thickness determines the contact area, which depends on the applied pressure. Figure 18d shows the multi-touch interface with 36 pixels of the pressure sensor. The proposed sensor can be integrated into the touch interface of a mobile device (e.g., smartphone).

**Figure 19:**
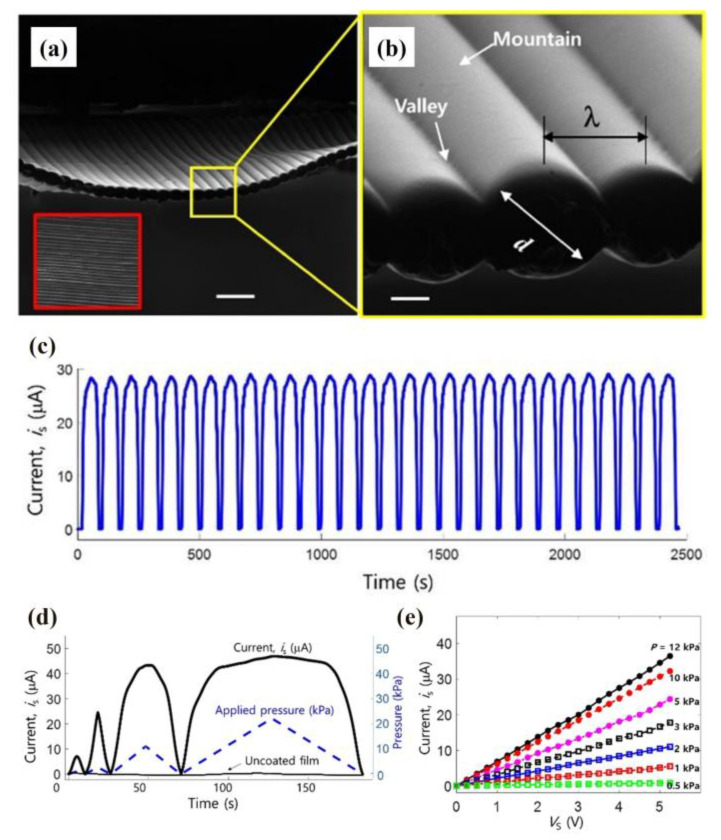
(**a**) SIM image and (**b**) magnified SIM image of highly ordered polyether block amide nanofibers. (**c**) Current response under the condition of a 200 Pa s^−1^ loading sweep rate. (**d**) Reversible loading and unloading behaviors of the pressure sensor under different peak pressures. (**e**) Different values of resistance at different applied pressures. Reprinted from ref. [71] with permission from IOP Publishing Ltd., copyright 2018.

Figure 19a shows the scanning ion microscopy (SIM) image of highly ordered polyether block amide nanofibers. The cylindrical microstructure presents a periodically ordered array and has uniform diameter distribution. Figure 19b shows the magnified SIM image of highly ordered polyether block amide nanofibers. Each electrospun nanofiber is closely connected and forms well-adhered interfaces. The diameter of each nanofiber is d, and the distance between the two nanofibers is λ. The highest point of each nanofiber is named ‘mountain’, and the lowest point between the two nanofibers is named ‘valley’.

Figure 19c shows the current response under the 200 Pa s^−1^ loading sweep rate condition. The sensor still has high sensitivity and repeatability after 2500 measurements. Figure 19d shows the pressure sensor’s reversible loading and unloading behaviors under different peak pressures. In Figure 19d, the blue dashed line represents the applied pressure—the applied pressure has four different peak pressures, which are 1, 5, 20 and 40 kPa. The black line above the blue dash line represents the temporal response of the pressure sensor with the coating layer, and the black line under the blue dash line represents it without the coating layer. Comparing the two lines, we can conclude that the sensor’s sensitivity with the coated film is drastically increased. Figure 19e shows the variations of the electrical resistance under various applied pressures. The reciprocal slope can calculate the different equivalent electrical resistance values of the sensor at different applied pressures.

This work demonstrates the potential application of this microstructure in pressure-sensing platforms. The ordered and compressible cylindrical microstructure is expected to be applied to flexible electronic products and wearable devices in the future.

In the past decade or so, researchers have made tremendous progress in the sensitivity, response time, and detection limits of pressure sensors. However, piezoelectric pressure sensors with electrospun nanofibers still face some challenges. (1) poor biocompatibility—among the current piezoelectric polymers, polyvinylidene fluoride (PVDF) and its copolymers have the highest piezoelectric performance, but their solvents are all environmentally hazardous; (2) Poor wear resistance—the wear resistance of electrospun nanofiber-based pressure sensors has not been well-focused and -characterized, though it is an important factor affecting the durability of wearable devices; (3) an immature electrospinning technique—the current electrospinning technology is not yet able to mass-produce nanofibers to prepare wearable devices.

Fortunately, electrospinning technology has made great progress in the past 20 years. The development of needleless electrospinning has significantly increased spinning material rates and yields. Near-field electrospinning enables the fabrication of neatly aligned fiber bundles and the construction of novel three-dimensional structures. It is expected that with the development of electrospinning technology, a new textile industry can be formed in the future to realize self-powered human health monitoring.

### 3.2. Humidity Sensor

**Figure 20:**
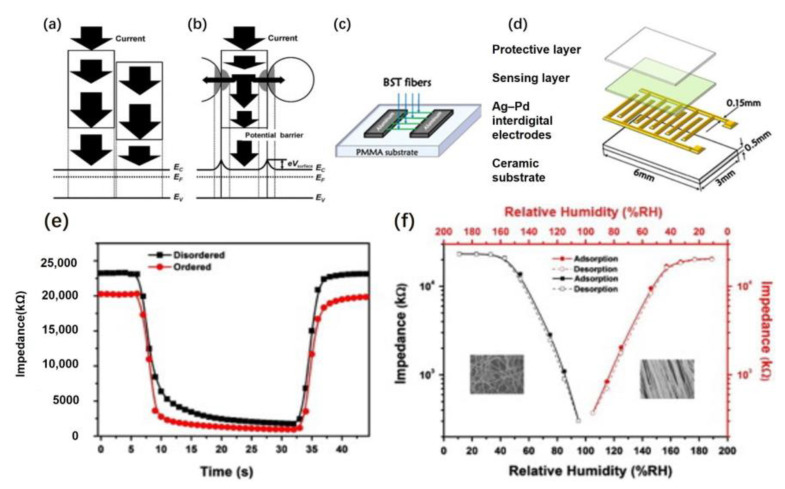
Schematic drawing of electron transmission in (**a**) disordered and (**b**) highly ordered nanofibers. (**c**) Collector device used to collect highly ordered BST fibers. (**d**) Schematic diagram of the humidity sensor. (**e**) Response–recovery properties of humidity sensors based on ordered and disordered fibers. (**f**) Humidity hysteresis characteristics using the ordered and disordered fibers. Reprinted from ref. [30] with permission from Elsevier BV, copyright 2012.

Xia et al. used electrospinning combined with electrical fabricated BST nanofiber arrays. Figure 20a,b shows the schematic drawing of electron transmission in disordered and highly ordered nanofibers. There are two routes for carrier transmission in fibrous materials. In disordered nanofibers, electrons are mainly transported axially along the fiber. In ordered nanofibers, electrons are mainly transported both axially and radially. Additionally, due to the radial quantum confinement effect, the carriers are mainly transported in the direction of the fiber axis. Figure 20c shows the collector device used to collect highly ordered BST fibers. The collector comprises two pieces of Al and a polymethyl methacrylate (PMMA) substrate. The gap between the two pieces of Al is 15 mm. Figure 20d shows the schematic diagram of the humidity sensor [30].

As seen in Figure 20e, the recovery time of the two materials is almost the same. Still, the response time of the ordered nanofiber is about 4.5 s faster than that of the disordered nanofiber. As shown in Figure 20f, the humidity hysteresis of the two fibers is the same. In conclusion, the response properties of ordered fibers were improved, but the recovery properties were almost unchanged compared with disordered fibers. The reason is that the ordered nanofiber array has more vital axial electron transport ability than the disordered nanofiber array does. Therefore, uniaxial aligned BST nanofibers have great application potential in preparing efficient humidity sensors.

This work has successfully synthesized uniaxially aligned BST nanofiber arrays. Although the response performance of ordered fibers is better than that of disordered fibers, there is no significant change in their recovery performance. Special attention needs to be paid to improving their recovery performance to manufacture more efficient humidity sensors. Because the synthetic-array humidity sensor shows rapid response and recovery at room temperature, the sensor is expected to be used to detect indoor humidity.

In addition to testing indoor environmental humidity, humidity sensors have broad application prospects in the production of flexible wearable devices. Zhou et al. [84] prepared ultra-flexible ionic fiber membranes using electrospinning technology. The membrane has both pressure- and humidity-sensing characteristics and can detect pressure and humidity simultaneously without them interfering with each other. Such multi-functional sensors have been successfully fabricated into smart bracelets and masks for pulse, skin moisture and breathing monitoring, which shows that humidity sensors also have potential applications in healthcare.

### 3.3. Strain Sensor

**Figure 21:**
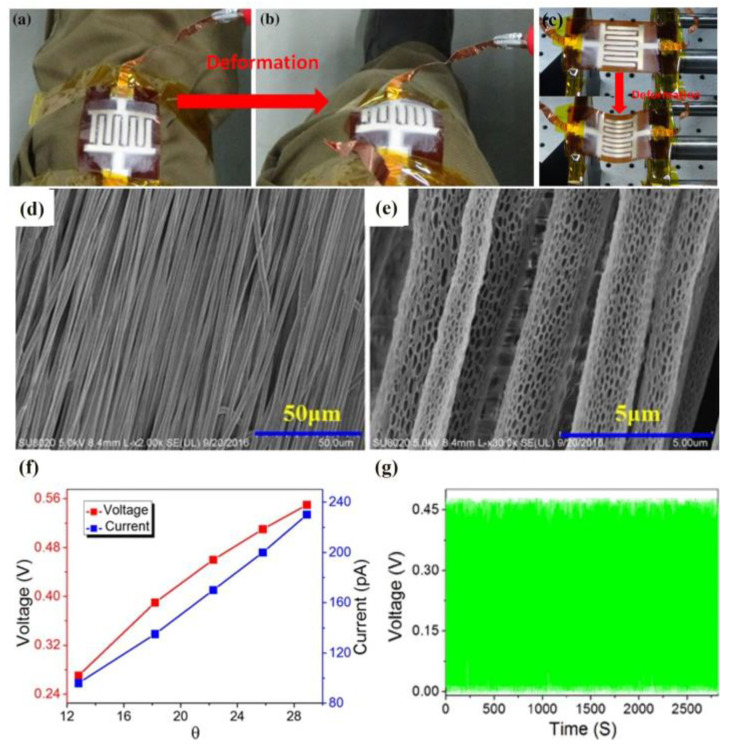
Simplified sensor with highly ordered nanofibers used for (**a**) the knee in the relaxed state and (**b**) the knee in the bent state. (**c**) Bending deformation during the test. SEM image of highly ordered PLLA nanofibers of diameters of (**d**) 50 μm and (**e**) 5 μm. (**f**) Relationship between peak short-circuit current, open-circuit voltage and deformation angle (θ). (**g**) Relationship between open-circuit voltage and measurement times. Reprinted from ref. [62] with permission from Springer Science Business Media New York, copyright 2017.

Zhu et al. [62] fabricated ordered, porous, piezoelectric PLLA nanofibers on comb electrodes via electrospinning. During direct current electrospinning, the electric dipole components on the central carbon chains of PLLA nanofibers can be polarized along the orientation direction. Figure 21a,b shows the simplified sensor with highly ordered nanofibers for human joints and tests. Periodic positive and negative accelerations with bending deformation are applied to the prototype for a periodic reciprocating motion, as shown in Figure 21c.

Figure 21d, e shows the SEM images of highly ordered PLLA nanofibers on different scales. PLLA nanofibers can be aligned along a direction because the electric dipole component along the main carbon chain can be polarized along the alignment direction. Figure 21f illustrates the relationship between the peak short-circuit current, open-circuit voltage and deformation angle (θ). Because the peak short-circuit current and open-circuit voltage have good linearity, the prototype can be used as a strain sensor. Figure 21g shows the open-circuit voltage after measuring it 2800 times. There is no decline after the long-term continuous operation, and the PLLA nanofiber prototype is reliable and durable [62].

In this work, ordered, porous, piezoelectric electrospun PLLA fibers were fabricated and strain sensors were prepared. The sensor is expected to be applied to the collection of energy generated during the movement of human joints. However, its maximum peak power is only 19.5 nW, which is far from that required for practical applications.

Many studies are conducted on preparing nanofibers and making strain sensors using electrospinning. Various materials with high flexibility and electrical properties are used, among which polymeric nanomaterials are the most promising. Electrostatically spun P(VDF-TrFE(Tri fluoroethylene)) nanofiber mats for 3D stress sensors have excellent sensitivity even at 0.1 Pa. Additionally, the aligned electrostatically spun core–shell P(VDF-TrFE) nanofibers are nearly 40 times more sensitive than thin-film PVDF-based stress sensors. PVDF and PVDF copolymer nanofibers produced by electrostatic spinning are expected to be the most promising materials for flexible tactile sensing applications.

### 3.4. Gas Sensor

**Figure 22:**
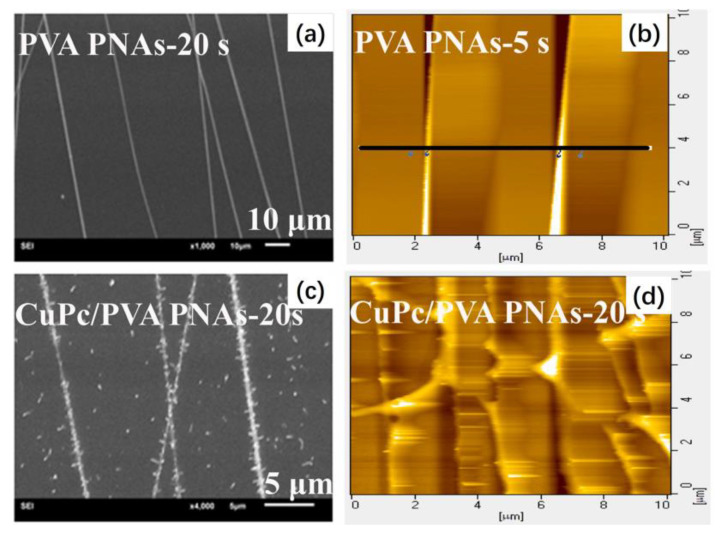
SEM images of (**a**) PVA parallel nanofiber arrays with an electrospinning time of 20 s and (**c**) CuPc/PVA parallel nanofiber arrays with an electrospinning time of 20 s. AFM images of (**b**) PVA parallel nanofiber arrays with an electrospinning time of 5 s and (**d**) CuPc/PVA parallel nanofiber arrays with an electrospinning time of 20 s. Reprinted from ref. [51] with permission from Elsevier BV, copyright 2021.

Wang et al. used an electrospun receiving board with an inclined gap and fabricated highly ordered PVA parallel nanofiber arrays. Then, they evaporated copper phthalocyanine (CuPc) on the PVA parallel nanofiber arrays to obtain a 3D CuPc organic field effect transistor sensor [51]. Figure 22a,b, respectively, shows the SEM and atomic force microscope (AFM) images of PVA parallel nanofiber arrays under the spinning times of 20 s and 5 s. The PVA parallel nanofiber arrays were aligned almost evenly on the substrates made of SiO_2_/Si, confirming that this receiving board could fabricate highly ordered PVA nanofibers. Figure 22c,d, respectively, shows the SEM and AFM diagrams of the CuPc/PVA parallel nanofiber arrays under a spinning time of 20 s. The CuPc crystal needles were acquired by evaporating the CuPc films on the PVA parallel nanofiber arrays. The results show that the films on the highly ordered PVA parallel nanofiber arrays and the SiO_2_ substrate worked together to form the 3D CuPc films.

**Figure 23:**
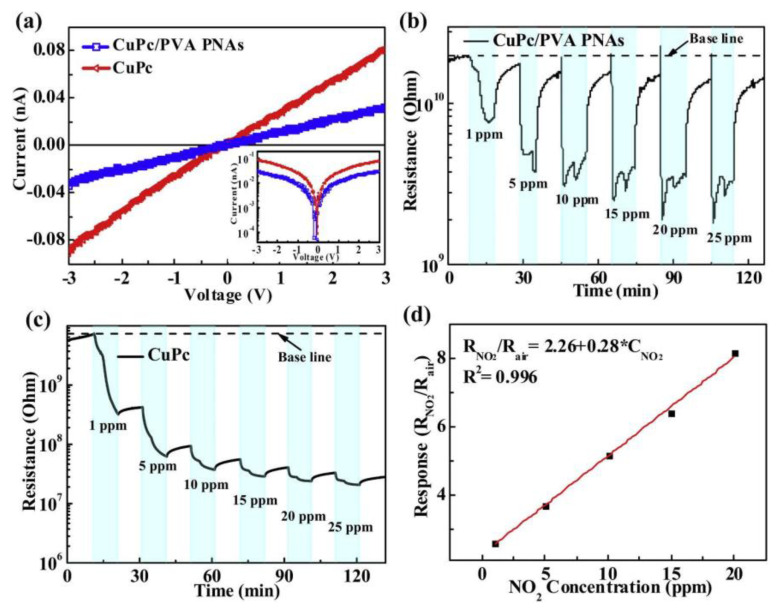
(**a**) The current–voltage curves of CuPc/PVA parallel nanofiber arrays and CuPc sensor. Typical dynamic responses of (**b**) CuPc/PVA parallel nanofiber arrays and (**c**) single CuPc sensors. (**d**) Relationship for CuPc/PVA parallel nanofiber arrays between NO_2_ concentration and its responses. Reprinted from ref. [51] with permission from Elsevier BV, copyright 2021.

Figure 23a shows the current–voltage curves of the CuPc/PVA parallel nanofiber arrays and the CuPc sensor. The illustration in the lower right-hand corner shows the logarithmic relationship between current and voltage. Figure 23a shows that the upper bound of the current values that can be measured is 1 × 10^−13^ A. The conductivity (slope) of the CuPc/PVA parallel nanofiber arrays sensor is lower than the conductivity of the single CuPc sensor. The reason for this may be that the CuPc/PVA sensor has thinner sensing films than the single CuPc sensor does under the same conditions. Figure 23b,c shows the typical dynamic responses of CuPc/PVA parallel nanofiber arrays sensor and of the single CuPc sensor. The initial sensor resistors of CuPc/PVA parallel nanofiber array sensors are 1.96 × 10^10^ Ω, and those of single CuPc sensors are 7.07 × 10^9^ Ω. The concentration of NO_2_ is changed from 1 ppm to 25 ppm, and the dotted line in the figure represents the baseline. As can be seen from the figures, the resistance of all sensors changed distinctly when the NO_2_ concentration was 1 ppm. Nevertheless, the recovery time of a single CuPc sensor is longer than that of a CuPc/PVA parallel nanofiber array sensor. At the same time, the recovery rate of a CuPc/PVA parallel nanofiber array sensor is much better than that of a single CuPc sensor. Figure 23d shows the relationship for CuPc/PVA parallel nanofiber arrays between the NO_2_ concentration and its responses. The results indicate that CuPc/PVA parallel nanofiber array sensors can detect the presence or absence of NO_2_ within a short time. It has a fast response/recovery property.

**Figure 24:**
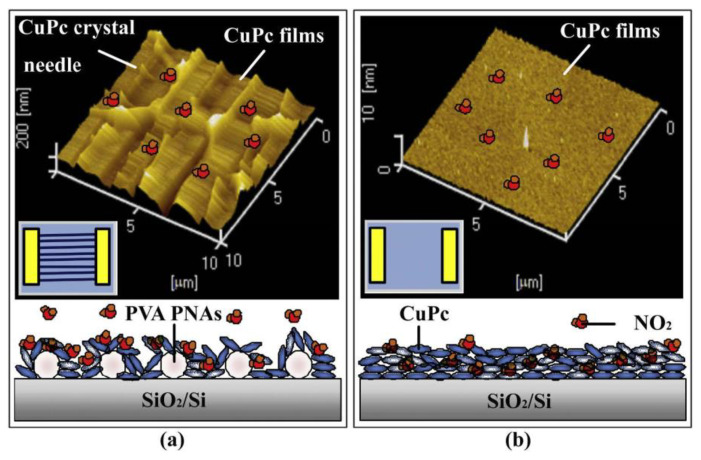
(**a**) AFM image (**top**) and lateral diagrammatic drawing of NO_2_ adsorption–desorption (**bottom**) of CuPc/PVA parallel nanofiber array films. (**b**) AFM image (**top**) and lateral diagrammatic drawing of NO_2_ adsorption–desorption (**bottom**) of single CuPc films. Reprinted from ref. [51] with permission from Elsevier BV, copyright 2021.

Figure 24 shows the AFM and schematic diagrams of CuPc/PVA parallel nanofiber arrays and single CuPc films. While the oxidizing NO_2_ is in contact with the surface of the CuPc films, the NO_2_ molecules are immediately absorbed on the film’s surface. Additionally, then, the NO_2_ molecules capture free electrons on the surface of 3D CuPc films with highly ordered PVA parallel nanofiber arrays and single CuPc films. Compared with single CuPc films, the 3D structure of CuPc films with highly ordered PVA parallel nanofiber arrays contact more NO_2_ molecules in various angles and directions. Therefore, the highly ordered PVA parallel nanofiber array sensor has broader concentration detection abilities.

In this work, highly ordered PVA parallel nanofiber arrays were spun, and sensors were prepared. The sensor can detect 0.3 ppm of NO_2_ gas and is expected to expand to the real-time detection of other gases.

Based on the environmental pollution caused by various types of gases and people’s requirements for quality of life, the future direction of research on gas sensors can be carried out in terms of the following aspects: (1) the development of gas sensors with excellent selectivity, ultra-high response, low detection limits and fast response times; (2) the combination of electrospinning with post-processing techniques such as chemical deposition or sputtering, which will result in nanomaterials with enhanced catalytic activity and attractive sensing properties; (3) The development of sensors that can provide good gas-sensitive properties at room temperature, as the optimum operating temperature of gas sensors is generally too high, and there is a risk of explosion in explosive environments. 

### 3.5. Biosensor

**Figure 25:**
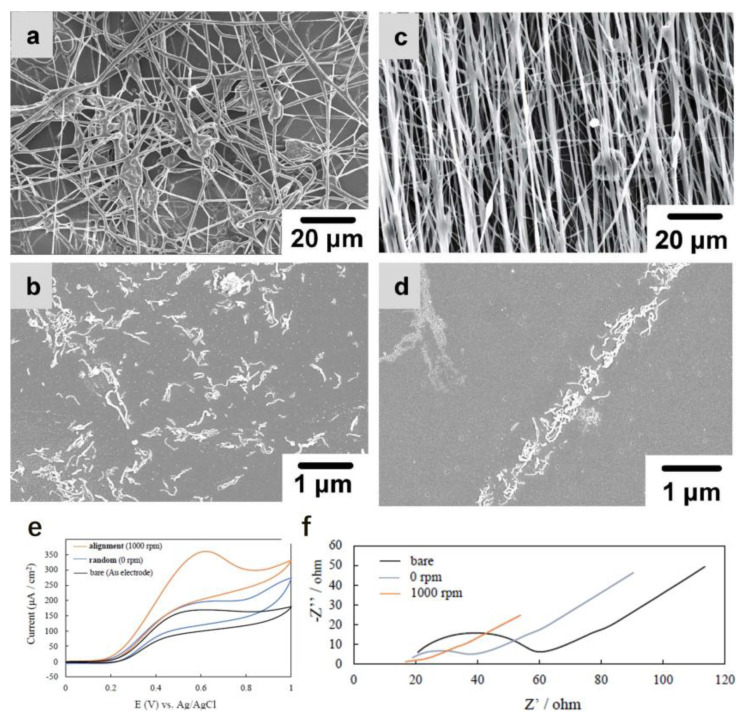
SEM images of PEVA/CNT fibers fabricated by (**a**) a still collector before combustion, (**b**) a still collector after combustion, a (**c**) rotated collector before combustion, and a (**d**) rotated collector after combustion. (**e**) Cyclic voltammograms of a Au/carbon nanotube/1—pyrenebutyric acid N—hydroxy succinimide ester/pyrroloquinoline quinone—dependent glucose dehydrogenase electrode and a bare Au electrode. (**f**) Impedance measurements with the Au/ordered carbon nanotube and Au/disordered carbon nanotube electrodes. Reprinted from ref. [85] with permission from Elsevier BV, copyright 2020.

Carbon nanotubes have the advantages of large specific surface areas and high electron transfer rates, which make them construct high-power biosensors. Nevertheless, van der Waals forces make carbon nanotubes aggregate easily and generate electrical resistance. Simply incorporating carbon nanotubes into electrodes directly cannot maximize its advantages. A technique was reported to obtain highly ordered carbon nanofibers to maximize their intrinsic conductive and structural properties. Figure 25a,c shows the SEM images of poly(ethylene-co-vinyl acetate)/carbon nanotube fibers collected by still and rotated collectors, respectively. A rotated collector can collect highly ordered nanofibers at 1000 rpm; the collector can still obtain disordered nanofibers at 0 rpm. Figure 25b,d shows the SEM images of poly(ethylene-co-vinyl acetate)/carbon nanotube fibers after combustion. Comparing the two images, it can be concluded that carbon nanotubes in the carbon nanotube-poly(ethylene-co-vinyl acetate) core–sheath nanofibers could be oriented by spinning the collector during electrospinning [85].

Figure 25e shows the cyclic voltammograms of Au/carbon nanotube/1-pyrenebutyric acid N-hydroxy succinimide ester/pyrroloquinoline quinone-dependent glucose dehydrogenase electrodes and bare Au electrode. From Figure 25e, we can know that the typical pyrroloquinoline quinone-dependent glucose dehydrogenase catalyzed oxidation current is significantly increased because of the addition of the glucose substrate. Figure 25f shows the impedance measurements with the Au/ordered carbon nanotube and Au/disordered carbon nanotube electrodes. The semi-circles refer to the charge transfer resistance of the particles. The radius of particles on the highly ordered carbon nanotube electrode (1000 rpm) is smaller than that on the unaligned carbon nanotube electrode (0 rpm). The results indicate that the highly ordered carbon nanotube electrode has a higher electron transfer efficiency on the electrode interface than it does on the unaligned carbon nanotube electrode.

In this work, pyrroloquinoline quinone-dependent glucose dehydrogenase highly ordered and disordered CNT nanofibers were prepared and made into electrodes. CV and impedance measurements show that ordered carbon nanotube electrodes perform better than random carbon nanotube electrodes do. This is expected to be valuable for building high-powered biological devices, such as biosensors and biofuel cells.

In the future, biosensors made using electrospun fibers may be developed in the following directions: (1) The diversification of functions. Biosensors will combine more functions in one. (2) The achievement of easy portability. Biosensors will be made more wearable and comfortable. (3) The achievement of intelligent and networked devices. Biosensors will connect with more smart devices and collect and analyze data in time.

## 4. Conclusions and Perspectives

This review overviews the highly ordered electrospun nanofibers in three aspects. Firstly, different electrospinning methods for forming highly ordered nanofibers re summarized. Electrospinning was modified to stabilize the polymer jet by introducing magnetic fields, controllable platforms and needles, and parallel electrodes. The improved collection devices can obtain highly ordered nanofibers. Secondly, different microstructures of highly ordered nanofibers are summarized and compared. Nanofibers are one-dimensional nanomaterials, but they can be arranged to form uniaxially ordered filaments, biaxially crossed and ordered nanofiber webs, and 3D-ordered scaffolds. Finally, different applications of highly ordered nanofibers in the field of sensors are summarized. Different nanofibers have attracted extensive attention because of their high specific surface area and reactive activity. Highly ordered nanofibers have been shown to have superior mechanical and dielectric strength compared to disordered nanofibers, which could expand their applications in the sensor field, such as in pressure sensors, humidity sensors, strain sensors, gas sensors and biosensors. 

Although many methods have been developed to fabricate highly ordered nanofibers, the preparation process still has some challenges and limitations: (1) Improvement of nanofiber production efficiency. Improvements in electrospinning equipment are needed to facilitate the transition from laboratory to industry. (2) Batch quality assurance. Fiber quality is greatly affected by environmental factors, and the change in temperature and humidity will bring different fiber quality. (3) The reduction of production cost. Including material and labor cost reduction. How to develop cheaper and more efficient production materials? (4) The limitations of characterization methods for nanomaterials. For example, SEM is commonly used to characterize the surface morphology of nanofibers. However, due to the electron beam secondary imaging principle of SEM, the surface morphology is easily incorrectly reflected. (5) The establishment of recognized characterization methods. For example, no recognized characterization method has been established for the strength and modulus of nanofibers.

## Data Availability

Not applicable.
